# Supplementary material for: Vitamin D Modified DSS-Induced Colitis in Mice via STING Signaling Pathway
Source: Biology (Basel). 2025 Jun 18;14(6):715. doi: 10.3390/biology14060715 (PMC12190092; doi:10.3390/biology14060715)
Supplement: Supplementary file 1 [file biology-14-00715-s001.zip › Supplementary Materials S1: 16s-report of gut microbiota/Groups/Lefse_Analysis/biomarkers_raw_images/1_d__Bacteria-p__Firmicutes-c__Bacilli-o__Lactobacillales-f__Enterococcaceae.pdf]

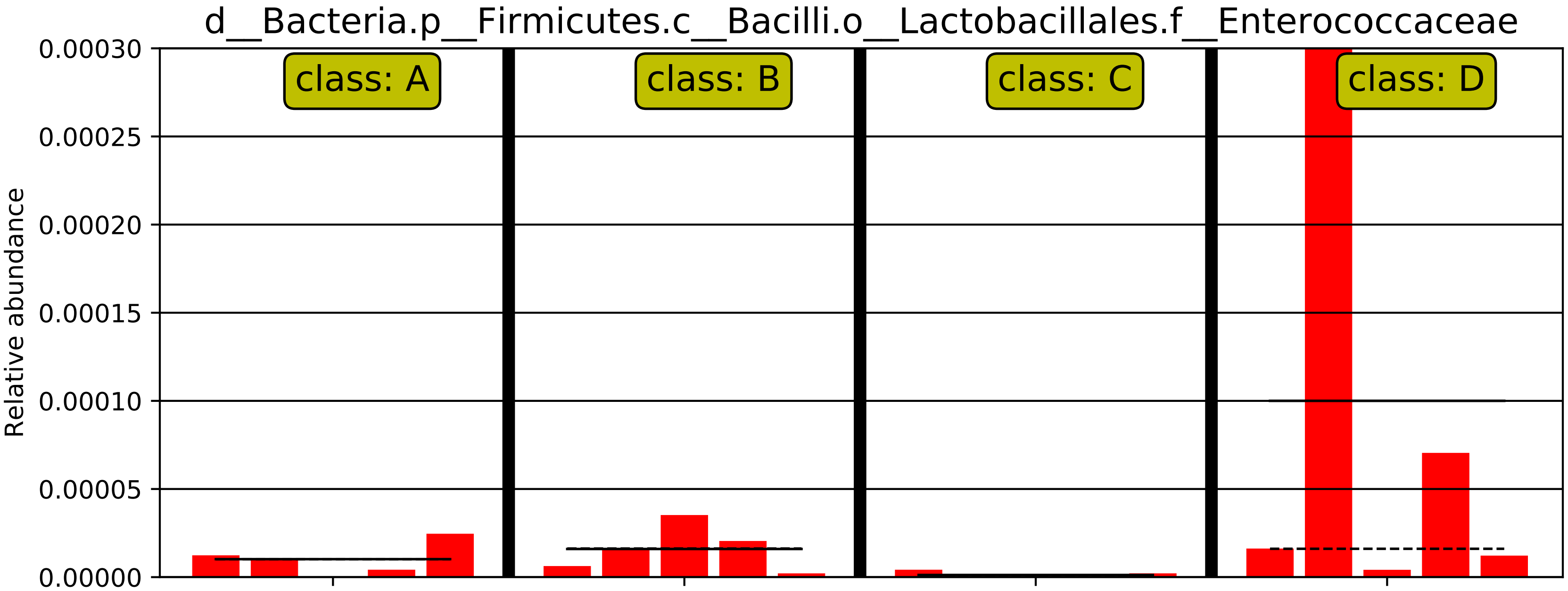
d__Bacteria.p__Firmicutes.c__Bacilli.o__Lactobacillales.f__Enterococcaceae
Relative abundance
0.00030
0.00025
0.00020
0.00015
0.00010
0.00005
0.00000
class: A
class: B
class: C
class: D
